# Ferroptosis in Neurological Diseases

**DOI:** 10.3389/fncel.2020.00218

**Published:** 2020-07-13

**Authors:** Jia-Xin Ren, Xin Sun, Xiu-Li Yan, Zhen-Ni Guo, Yi Yang

**Affiliations:** ^1^Department of Neurology, The First Hospital of Jilin University, Changchun, China; ^2^School of Clinical Medicine, Jilin University, Changchun, China; ^3^Clinical Trial and Research Center for Stroke, Department of Neurology, The First Hospital of Jilin University, Changchun, China

**Keywords:** ferroptosis, neurodegeneration, stroke, inhibitors, iron

## Abstract

Ferroptosis is mechanism for non-apoptotic, iron-dependent, oxidative cell death that is characterized by glutathione consumption and lipid peroxides accumulation. Ferroptosis is crucially involved in neurological diseases, including neurodegeneration, stroke and neurotrauma. This review provides detailed discussions of the ferroptosis mechanisms in these neurological diseases. Moreover, it summarizes recent drugs that target ferroptosis for neurological disease treatment. Furthermore, it compares the differences and relationships among the various cell death mechanisms involved in neurological diseases. Elucidating the ferroptosis role in the brain can improve the understanding of neurological disease mechanism and provide potential prevention and treatment interventions for acute and chronic neurological diseases.

## Ferroptosis

Ferroptosis is a recently identified type of regulated cell death (Dixon, 2017). Specifically, it is a non-apoptotic, iron-dependent, oxidative cell death mechanism that was proposed by Dixon et al. (2012). Ferroptosis, which is classified as “cell sabotage” (Green and Victor, 2012), differs from apoptosis, necrosis, and autophagy in terms of morphology, biochemistry, and heredity (Cao and Dixon, 2016). Ferroptosis is mainly characterized glutathione (GSH) consumption and lipid peroxidation, which involves specific oxidation of phosphatidylethanolamine containing arachidonic and adrenal acids (D’Herde and Krysko, 2017). The following unique gene set is required for erastin-induced ferroptosis: ribosomal protein L8, iron response element-binding protein 2, F0-F1-ATPase subunit C3, citrate synthase, tetratricopeptide repeat domain 35, and acetyl-CoA synthetase family member (Speer et al., 2013).

Ferroptosis is studied through step-wise *in vivo* and *in vitro* experiments using various activators and inhibitors (Xie et al., 2016). Previous cell experiments (Dixon et al., 2012; Yang et al., 2014; Do Van et al., 2016) have shown that ferroptosis is triggered by the oncogenic RAS-selective lethal small molecule erastin, RAS-selective lethal 3, buthionine sulfoximine and sulfasalazine. Ferroptosis induced by the aforementioned compounds and drugs has been detected in cancer cells (Yagoda et al., 2007), kidney tubule cells (Friedmann Angeli et al., 2014), neurons (Guiney et al., 2017), and T cells (Matsushita et al., 2015). Consequently, there are various types of ferroptosis inhibitors with different targets including iron chelators (Ward et al., 2014), antioxidants (Dixon et al., 2012), ferrostatins-1 (Fer-1) (Skouta et al., 2014), glutathione peroxidase 4 (GPX4) (Yang et al., 2014), hypoxia-inducible factor prolyl hydroxylase inhibitors (Speer et al., 2013) and other Chinese medicine (Andersen et al., 2019). Trials on these drugs have shown that the ferroptosis mechanism involve lipid reactive oxygen species (ROS) production, plasma membrane polyunsaturated fatty acid (PUFA) oxidation, iron metabolism, and antioxidant GSH metabolism (Ward et al., 2014; Kagan et al., 2017; Wu et al., 2018). Notably, ferroptosis is not cell suicide; rather it involves cell sabotage that occurs normal life activities to adapt to stimuli and body changes (Green and Victor, 2012; Dixon, 2017). Therefore, ferroptosis is only observed in multiple physiological and pathological processes, including cancer cell death, neurotoxicity, neurodegenerative diseases, acute renal failure, drug-induced hepatotoxicity, hepatic and heart ischemia/reperfusion injury, and T-cell immunity (Xie et al., 2016), when it is misregulated or unbalanced where it induces other cell death mechanisms and immunoreactions (Wenzel et al., 2017; Martin-Sanchez et al., 2018; Proneth and Conrad, 2019).

Ferroptosis plays an important role in the brain and neurological diseases (Kenny et al., 2019). The brain has the highest levels of PUFAs, which are recognized as lipid peroxide precursors, in the human body (Bazinet and Layé, 2014). Moreover, there is a close to correlation of GSH depletion and lipid peroxidation with neurological diseases, including neurotrauma, stroke, and neurodegeneration (Bayir et al., 2006; Weiland et al., 2019). Studies have reported an increase in the ferroptosis phosphatidylethanolamine (PE) marker after traumatic brain injury (TBI) (Wu et al., 2019). Moreover, cellular ferroptosis has been observed in dopaminergic neurons in Parkinson’s disease (PD) and other neurodegenerative diseases (Do Van et al., 2016). Similarly, ferroptosis inhibitors, including Fer-1 and liproxstatin-1 (Lip-1), have been shown to significantly reduce functional deficits in ischemic stroke mouse models. This indicates that ferroptosis is involved in stroke and ischemia-reperfusion injury (Tuo et al., 2017). Further, GPX4 genetic defects have been observed in *in vivo* and *in vitro* neuronal death (Yoo et al., 2012; Chen et al., 2015). Taken together, ferroptosis is a potential therapeutic target for neurological diseases given the complexity and fine regulation of the central nervous system (CNS), as well as the unclear changes in neurological diseases.

The targeted ferroptotic mechanism depends on the pathogenesis of the neurological disease; however, lipid peroxidation is generally regarded as the driving force of ferroptosis (Yang and Stockwell, 2016). Hence, we first introduce lipid peroxidation followed by a summary of the main ferroptotic mechanisms and drugs targeting ferroptosis to treat neurodegenerative diseases, stroke, and brain trauma.

## Lipid Peroxidation Is the Driving Force of Ferroptosis

Lipid peroxidation products are recognized as the most potent inducers of ferroptosis (Kagan et al., 2017). Lipid peroxide biosynthesis can be performed by enzymes, including 12/15-lipoxygenase (12/15-LOX) and 5-lipoxygenase (5-LOX), or through non-enzymatic processes known as Fenton-type chemistry (Gaschler and Stockwell, 2017). Lipid peroxidation is preferential for PUFAs, which are long-chain fatty acids with more than one double bond, including linoleic, arachidonic, and docosahexaenoic acids. They are highly enriched in the brain where they increase membrane fluidity and plasticity to promote neurotransmitter release and neural network development and migration (Gaschler and Stockwell, 2017; Ingold et al., 2018).

**FIGURE 1:**
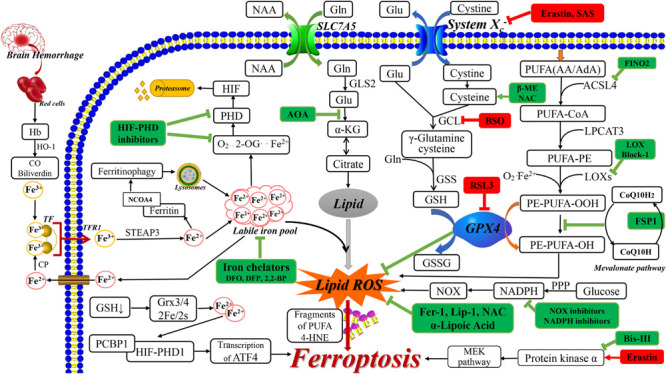
The related ferroptosis mechanisms in neurological diseases. The drugs with red box are ferroptosis inducer and the drugs with green box are ferroptosis inhibitor. AA, arachidonic acid; AdA, adrenic acid; ACSL4, acyl-CoA synthetase long-chain family member 4; AOA, amino acetate; α-KG, α ketoglutarate; BSO, buthionine sulfoximine; 2,2-BP, 2,2-bipyridyl; β-ME, β-mercaptoethanol; CO, carbon monoxide; CoQ 10, Coenzyme Q10; CP, ceruloplasmin; DMT1, divalent metal transporter 1; FAC, ferric citrate; FINO2, 1, 2-dioxolane; FSP-1, Ferroptosis-suppressor-protein 1; GCL, glutamate cysteine ligase; GLS2, glutaminase 2; Glu, glutamate; GSS, glutathione synthetase; GSSG, oxidized GSH; Hb, hemoglobin; HIF, hypoxia-inducible factor; 4-HNE, 4-hydroxynonenal; HO-1, heme oxygenase 1; Lip-1, liproxstatin-1; LPCAT3, lysophosphatidylcholine acyltransferase 3; NAA, neutral amino acids; NAC, N-acetylcysteine; NOX, nitrogen oxide; 2-OG, 2-oxoglutarate; PPP, pentose phosphate pathway; RSL3, RAS-selective lethal 3; SAS, sulfasalazine; STEAP3, 6-transmembrane epithelial antigen of the prostate 3.

Lipid peroxidation is inhibited by GPX enzymes, especially GPX4, which reduces phospholipid hydroperoxide to lipid alcohols (Brigelius-Flohé and Maiorino, 2013) and restricts the formation of reactive lipid alkoxy groups (Seiler et al., 2008). Lipid peroxide-induced ferroptosis involves the following three steps shown in Figure 1. First, as a key regulator, acyl-CoA synthetase long-chain family member 4 (ACSL4) catalyzes the esterification of arachidonoyl (AA) or adrenoyl (AdA) into PE. Second, lysophosphatidylcholine acyltransferase 3 (LPCAT3) is specific for PE-based substrates and generates PUFA-PE. Finally, 15-LOX oxidizes AA-PE and AdA-PE into ferroptotic signals, including PE-AdA-OOH and PE-AA-OH (D’Herde and Krysko, 2017). For high lipid peroxide levels, lipid peroxide aggregates have been reported in the endoplasmic reticulum (Doll et al., 2017). Moreover, other studies have shown that lipid peroxidation accumulation on the mitochondrial membrane is a key factor for ferroptosis (Friedmann Angeli et al., 2014; Krainz et al., 2016). Here, CRISPR-based genome-wide genetic screening and microarray analysis have demonstrated ACSL4 and LPCAT3 as key ferroptosis regulators (Dixon et al., 2015; Doll et al., 2017). Notably, GPX4 depletion induces ferroptosis only when ACSL4 and LPCAT3 are expressed and involved in arachidonic acid modification with subsequent insertion into membrane phospholipids (Cheng and Li, 2007; Shindou and Shimizu, 2009). Downstream events of lipid peroxides include PUFA fragmentation and membrane lipid damage, as well as toxic reactive lipid intermediate 4-hydroxynonenal production. This leads to cellular ferroptosis promotion through covalent modification and inactivation of essential intracellular proteins (Schneider et al., 2008).

Studies using erastin have shown that system Xc^–^, which is a transmembrane cystine/glutamate reverse transporter, plays an important role in ferroptosis (Dixon et al., 2012). System Xc^–^ is comprised of the 12-pass transmembrane transporter SLC7A11 (Sato et al., 1999), which promotes cysteine-dependent GSH synthesis (Dixon et al., 2012). The following steps lead to ferroptosis. First, erastin inhibits system Xc^–^ and competitively inhibits cystine uptake, which leads to cysteine depletion (the rate-limiting precursor for GSH synthesis). Subsequently, this causes GSH depletion, which leads to imbalanced cellular oxidants and antioxidants, and oxidative, which induces cell death (Siddiq et al., 2005; Figure 1). Furthermore, A Gln- and citrate synthase-dependent lipid synthesis pathway can supply specific lipid precursors required for ferroptosis (Dixon et al., 2012).

## Ferroptosis in Neurodegeneration

### The Ferroptosis Mechanism in Neurodegeneration: Free Iron Accumulation

Neurodegenerative diseases include complex and elaborate cell death mechanisms and multiple pathways, which involve excessive accumulation of iron and lipid peroxidation in different brain regions (Guiney et al., 2017). An important feature of nervous system dysfunction is the metabolic and nutritional coupling between glial cells (astroglial cells, oligodendrocytes, and microglia) and neurons, which can lead to neuronal death, especially in ferroptosis (Ratan, 2020). Ferroptosis is dependent on excessive iron accumulation, which is a crucial component of lipid oxidation and is derived from iron metabolism disorder (Cao and Dixon, 2016). Under normal CNS conditions, iron is mainly combined with ferritin and neuromelanin. Iron, which is an important cofactor in the CNS (Moreau et al., 2018), participates in several important processes, including oxygen transportation, oxidative phosphorylation, myelin production, and neurotransmitter synthesis and metabolism (Ward et al., 2014). Through the transferrin-transferrin receptor 1 (TF-TFR1) system, iron is released into the cytoplasmic after crossing the blood-brain barrier. Subsequently, it continuously moves among neurons, oligodendrocytes, and astrocytes. The inactive iron (Fe^3+^) form is recognized by TF and moved into the cell by TFR1. Subsequently, Fe^3+^ is converted into free iron (Fe^2+^) by 6-transmembrane epithelial antigen of the prostate 3 (STEAP3) (Ke and Qian, 2007; Wu et al., 2019). Free iron is transported by divalent metal transporter 1 (DMT1) from the endosome and exported by ferroportin. Free iron can be stored in ferritin or the labile iron pool with the latter being involved in lipid ROS generation. Fe^2+^ is a cofactor for metalloenzymes in many oxidation reactions, including those modulated by lipoxygenase and hypoxia-inducible factor prolyl hydroxylase (HIF-PHD) (Karuppagounder and Ratan, 2012; Kagan et al., 2017; Yan and Zhang, 2019). Further, free iron can be released to the labile iron pool by lysosomes, which degrade ferritin via nuclear receptor coactivator 4 (NCOA4)-mediated ferritinophagy (Torii et al., 2016). Therefore, brain iron homeostasis is maintained through the regulation of iron movement between blood and brain tissue; intracellular and extracellular; and among different iron pools (Ke and Qian, 2007; Garton et al., 2016). Protein-bound iron is safe; contrastingly, abnormal iron homeostasis involving excessive free iron generates molecules that can destroy proteins and nucleotides through Fenton chemical reactions, which leads to lipid peroxidation (Ke and Qian, 2007; Wu et al., 2019). Therefore, iron is intricately involved in the onset and progression of neurodegenerative diseases (Siddiq et al., 2005; Ward et al., 2014). The involvement of excessive iron accumulation in neurodegenerative diseases includes oxidative stress induction, mitochondrial function interference, excessive ROS production, and destruction of nuclear DNA and mitochondria (Ke and Qian, 2007). Daniel I Orellana et al. (2016) have shown that in neurodegenerative diseases, pantothenate kinase-associated neurodegeneration may disrupt lipid homeostasis, change the mitochondrial iron-dependent biosynthetic pathway and cytoplasmic iron homeostasis and produce harmful ROS. Moreover, iron accumulation in neurodegenerative diseases could involve gene mutations related to iron transport and binding, changes in the TF transport system, increased divalent metal transporter 1 expression (Cheah et al., 2006), and blood-brain barrier dysfunction (Ward et al., 2014; Figure 1).

### Ferroptosis Inhibitors in PD

#### Ferroptosis Induction by Excess Iron in PD

PD is characterized by neuronal loss in multiple brain regions, especially dopaminergic neurons in the substantia nigra. Moreover, the causative factor of PD is α-synuclein protein (Guiney et al., 2017). Based on these PD traits, the key treatment target is restoring brain dopamine levels and preventing neuron degeneration. Iron-dependent ferroptosis is a crucial cell death pathway for dopaminergic neurons (Do Van et al., 2016). In PD, dopaminergic neurons have abundant iron, which is an integral part of enzymatic and non-enzymatic reactions involved in dopamine metabolism (Moreau et al., 2018). In addition, free iron induces dopamine oxidation and α-synuclein aggregation (Duce et al., 2017) which leads to increased dopaminergic neuron loss in PD; moreover, it mutates TF and causes excessive iron uptake (Ward et al., 2014). The possible underlying mechanism could involve excess free iron being able to reduce GPX4 activity, which, in turn, leads to GSH depletion. Subsequently, there is a reduced ROS clearance ability of cells, which leads to excessive ROS, membrane oxidation, and eventual ferroptosis (Imai et al., 2017; Stockwell et al., 2017; Wang et al., 2017). Moreover, α-synuclein oligomers significantly increase the rate of active oxygen production, and induce lipid peroxidation and the increase of iron-dependent free radicals in neurons and astrocytes, such aggregates will be incorporated into the cell membrane through mutual action leading to ferroptosis, which may play an important role in the cellular mechanism of neuronal cell loss in Parkinson’s disease (Angelova et al., 2015, 2020). Experiments show that PUFA is more resistant to lipid peroxidation through site-specific deuteration, which can prevent α-Syn-induced lipid peroxidation and α-Syn-induced cell death (Angelova et al., 2015).

#### Iron Chelators in PD

Iron chelators are ferroptosis inhibitors, that target free iron in labile iron pools and prevent iron from supplying electrons to oxygen to form ROS (Dixon and Stockwell, 2014). There are two categories of iron chelators, membrane-permeable (lipophilic iron chelators), and membrane-impermeable (Cao and Dixon, 2016). Lipophilic iron chelators, which include ciclopirox and 2,2-bipyridyl, penetrate the cell membrane and blood-brain barrier, target iron accumulation areas, and remove chelatable iron from labile iron pools or transport it to other proteins (Boddaert et al., 2007). For ferroptosis inhibition, lipophilic iron chelators prevent PUFA fragment oxidation and lipid ROS formation (Gao et al., 2015). Moreover, lipophilic iron chelators can directly inactivate ferrous enzymes that promote membrane lipid oxidation (Cao and Dixon, 2016), as well as hypoxia-inducible factor-prolyl hydroxylases (Siddiq et al., 2005). This exerts neuroprotective effects by simulating hypoxia, stabilizing HIF-1α, and activating hypoxia-adaptive genetic programs (Karuppagounder and Ratan, 2012). In contrast, membrane-impermeable iron chelators include deferoxamine (DFO) and deferiprone (DFP), which accumulates in lysosomes through endocytosis from where they capture excess free iron (Barradas et al., 1989).

Numerous *in vivo* and *in vitro* experiments have shown that iron chelators play an essential role in PD (Do Van et al., 2016). DFP reduces oxidative stress, increases cellular dopamine utilization, improves existing motor symptom, and prevents worsening in MPTP mouse models (Devos et al., 2014). A phase III clinical trial on iron chelators treatment in patients with PD reported reduced dyskinesia and increased GSH peroxide activity in the cerebrospinal fluid within 6 months (Guiney et al., 2017; Martin-Bastida et al., 2017). Notably, most patients with neurodegenerative disease present normal systemic iron homeostasis during iron chelators application (Ward et al., 2014). Excessive systemic iron chelation therapy is unsuitable for patients with PD and may cause iatrogenic iron depletion and anemia. Therefore, low-dose iron chelation therapy should be used to allow neuroprotective effects with minimal side effects (Moreau et al., 2018). A novel strategy based on iron clearance and redeployment targets has been proposed to allow targeted and differential specificity (Cabantchik et al., 2013). A study conducted by Devos et al. (2014) administered 30 mg/10 g/day DFP to patients with PD. Clinical benefits were observed at 6 months, dyskinesia was significantly reduced at 12 months, and a normal iron index was restored at 18–24 months. This indicated that a lower DFP dose was safe with a risk for reversible neutropenia of 1–3% (Devos et al., 2014). Moreover, a study by Zhang et al. (2020) reported that ferroptosis occurred at a relatively low ferric citrate concentration, while apoptosis occurred with an increase in the iron dose. This is indicative of the role of early death in PD and shows that ferroptosis can result in apoptosis caused by iron overload. Furthermore, the specific ferroptosis inhibitor Fer-1 and the iron chelator DFO inhibit iron-induced cell death and apoptosis in the early PD stages (Zhang et al., 2020). In addition, a recent study shows that in the rodent Parkinson’s disease model, Clioquinol has the effect of an iron chelator. By reducing the iron content in substantia nigra, it inhibits the production of lipid peroxides and ferroptosis, and produce a therapeutic effect (Shi et al., 2020).

### Ferroptosis Inhibitors in Alzheimer’s Disease (AD)

#### The Relationship Between Ferroptosis and AD

AD is characterized by progressive cortical and hippocampal neuronal dysfunction and death. Its main pathological changes include hyperphosphorylation, abnormal Tau aggregation, and synapses and neurons degeneration (Dugger and Dickson, 2017; Roubroeks et al., 2017); synapses are lost and neuronal death is the underlying AD cause (Hambright et al., 2017). Ferroptosis characteristics (iron dysregulation, lipid peroxidation, and inflammation) are considered as important preclinical signs of AD and cognitive impairment (Chen et al., 2015; Hambright et al., 2017). Lipid peroxidation is considered an early event in AD pathogenesis (He et al., 2003). Specifically, excess iron aggravates toxic amyloid β peptide and hyperphosphorylated tau aggregation; moreover, it directly induces oxidative neuronal damage (Thirupathi and Chang, 2019). Iron interacts with amyloid β and tau through peptide-heme complex formation, and thus contributes to ROS generation, which might be involved in the ferroptosis pathway (Derry et al., 2020). Moreover, brain tissues from patients with AD have increased levels of 4-hydroxynonenal, a lipid oxidation by-product, which contributes to β-amyloid accumulation (Yoo et al., 2010).

#### Lipid Peroxidation Inhibitors in AD

A study by Zhang Y. H. et al. (2018) reported that an AD mouse model with Tau overexpression and hyperphosphorylation presented with excess iron. Studies have proposed treatment with α-lipoic acid (LA), which has natural enzyme cofactors with antioxidants and iron chelator properties; moreover, it easily penetrates the blood-brain barrier. LA administration has been shown to down-regulate TFR expression and up-regulate ferroportin 1expression, which reduces the iron overload. LA regulates iron redistribution through iron chelation; moreover, it acts as a direct free radical scavenger and an indirect antioxidant. Therefore, it reduces ROS levels and increases antioxidant enzymes expressions (GPX4 and Xc^–^) to play the neuroprotective role in AD (Persson et al., 2003). Furthermore, GPX4-knockout mice present cognitive impairment and neurodegenerative changes, which are associated with lipid peroxidation, extracellular signal-regulated kinase activation, and markedly increased neuroinflammation (Hambright et al., 2017). Therefore, GPX4 protects cortical neurons from oxidative damage and amyloid toxicity (Ran et al., 2006; Yoo et al., 2010). Notably, hydroxylated chalcone inhibits Aβ aggregation and ferroptosis by exerting a pharmacological effect on factors for iron-induced death (including GPX4 or Xc^–^ inhibition); therefore, it could be a candidate treatment for AD (Cong et al., 2019).

### Others

Bersuker et al. (2019) used synthetic lethal CRISPR/Cas9 screening technology, and the results showed that myristoylation recruited ferroptosis-suppressor-protein 1(FSP1) to the plasma membrane, which acts as an oxidoreductase, reducing coenzyme Q10, producing a lipophilic free radical trapping antioxidant to prevent the spread of lipid peroxides. The FSP1-CoQ 10 -NAD(P)H pathway exists as an independent parallel system and can be used in conjunction with GPX4 and glutathione to inhibit phospholipid peroxidation and ferroptosis (Bersuker et al., 2019; Doll et al., 2019). Virus-inactivated and heat-treated human platelet lysate has a strong neuroprotective effect against erastin-induced ferroptosis (Nebie et al., 2019) and has been developed as a novel and effective neurodegenerative therapy. Human platelet lysates can inhibit ferroptosis and reduce neuronal loss in cellular models of PD and amyotrophic lateral sclerosis (ALS) (Gouel et al., 2017). Third, as another type of inhibitor, Mithramycin, DNA-binding drugs, can reduce c-Myc expression by binding to the Sp1 site in its promoter, thereby playing a role in inhibiting iron death and treating animal models of HD and AD (Sleiman et al., 2011; Ratan, 2020). Fourth, in mouse hippocampal neuron cells, gastrodin inhibits glutamate-induced ferroptosis through the Nrf2/HO-1 signaling pathway (Jiang et al., 2020). Besides, Nrf-2 activators (such as Tecfidera) are now used in clinical applications to reduce the exacerbation of multiple sclerosis involving ferroptosis and see hope in other neurodegenerative diseases (Sejbaek et al., 2019). Fifth, it is worth noting that diacetyl-bis (4-methyl-3-thiosemicarbazonato) copper II [Cu II (atsm)], by inhibiting ferroptosis, delays the progression of ALS and PD. Compared with lipoxstatin-1 (which has similar potency *in vitro*), Cu II has better properties including oral bioavailability and entry into the brain (Southon et al., 2020). Moreover, Vitamin E/α-tocopherol could act as a 12/15-LOX inhibitor in neurodegenerative diseases (Matsushita et al., 2015). Finally, it was also found that the transglutaminase inhibitor Cystamine can eliminate glutamate-induced ferroptosis (Farrelly et al., 2019) and the histone deacetylase inhibitor PCI-34051 is an effective neuroprotection and has a role in preventing neuronal ferroptosis (Sleiman et al., 2014).

## Ferroptosis in Stroke

### Ferroptosis in Ischemic Stroke

#### The Mechanism of Ferroptosis in Ischemic Stroke

Hypoxia-inducible factor prolyl hydroxylases (HIF-PHD) is considered as a neuroprotective target for “antioxidant” metal chelators. PHD, which is the main oxygen sensor, promotes HIF decomposition and inhibition during the normoxic state. However, PHD inhibition during the hypoxia condition maintains HIF stability (Karuppagounder and Ratan, 2012). On the one hand, HIF-PHD is involved in iron-chelator-related mechanism. Specifically, iron chelator inhibits HIF-PHD activity, which inhibits hydroxyl group production. Consequently, unhydroxylated HIF-1α does not bind ubiquitin-protein ligase and is not degraded. As a result, the [HIF-1α]-[HIF-1β] heterodimer activates protective gene expression, including vascular endothelial growth factor and erythropoietin. This exerts further neuroprotective effects and improves acute stroke recovery (Semenza et al., 2000; Bruick, 2003; Siddiq et al., 2005). On the other hand, the possible process of HIF-PHD causing ferroptosis is as follows: when glutathione is depleted, iron released from the disruption of the glutathione-Grx3/Grx4 Fe/S cluster complex could then be taken up into iron chaperones (PCB1) for enzymes such as the HIF PHDs, and then drive the expression of iron-promoting ATF4 gene, thereby causing ferroptosis (Ratan, 2020).

#### Ferroptosis Inhibitors in Ischemic Stroke

Ischemic stroke is characterized by rapid neuronal death and dysfunction. Moreover, post-ischemic stroke complications cause further neuronal damage. Therefore, there is a need for further research to further improve stroke prevention and treatment (Tuo et al., 2017). HIF-PHD inhibitors have been shown to reduce neuronal damage caused by permanent focal ischemia and can be clinically used as neuroprotective agents in cases with an imminent risk of ischemic or oxidative neuron damage (Siddiq et al., 2005). In addition, dimethyloxalylglycine significantly reduced the infarct volumes and improved behavior at 24 h and 8 days. Moreover, it improved regional cerebral blood flow after 24 h in a rat model with permanent and transient middle cerebral artery occlusion (Nagel et al., 2011).

A feasible strategy for first-line stroke treatment 12/15-LOX is inhibition using LOXBlock-1, which protects neuronal HT22 cells from oxidative stress. In a mouse model of transient focal ischemia, LOXBlock-1 reduced the infarct size and improved behavioral parameters at both 24 h and 14 days after stroke. This protective effect was observed even with delayed treatment until at least 4 h after the ischemic attack. Additionally, since LOXBlock-1 showed no adverse effects in the collagenase-induced bleeding model, it could be used as an adjuvant for tissue plasminogen activator thrombolysis (Yigitkanli et al., 2013). Notably, intraperitoneal Tat Selpep injection to induce GPX4 expression can reduce the post-focal ischemia infarct size. Moreover, in the mouse ischemic model, it drives the transcription reaction to offset reactive lipid formation and cell death, as well as improves important nerve function within clinically relevant time windows (Alim et al., 2019). Intranasal Lip-1/Fer-1 administration to middle cerebral artery occlusion mice significantly alleviated MCAO-induced functional defects and reduced the infarct size after 14 days (Tuo et al., 2017). Analysis of the drug structure indicated that “amines” are essential for inhibition of cellular ferroptosis; further, Fer-1 analog synthesis can develop more efficient ferroptosis inhibitors, including SRS11-92, SRS12-45, etc. (Skouta et al., 2014).

### Ferroptosis in Hemorrhagic Stroke

#### The Mechanism of Ferroptosis in Hemorrhagic Stroke

Post-hemorrhagic stroke damage is triggered by red blood cells lysis; hemoglobin, heme, and iron release; and coagulation cascade activation. This leads to irreversible destruction of neurovascular unit components with subsequent blood-brain barrier destruction, extensive neuronal death, and fatal brain edema (Qureshi et al., 2009; Keep et al., 2012; Zhao et al., 2018). After intracranial hemorrhage (Garton et al., 2016), heme is degraded by heme-oxygenase into carbon monoxide, biliverdin, and free iron, which induces ferroptosis (Wu et al., 2019; Figure 1).

#### Ferroptosis Inhibitors in Hemorrhagic Stroke

In the hemorrhagic stroke model, Tat Selpep effectively drives GPX4 expression in the brain, protects and protect neurons, and improves behavior (Alim et al., 2019). Adaptaquin reduces cell death and enhances functional recovery in intracerebral hemorrhage (ICH) rodent models (Karuppagounder et al., 2016). Through toxic lipids neutralizations, the synergistic effect of clinically approved thiol-containing redox-regulating N-acetylcysteine and prostaglandins E inhibits heme-induced ferroptosis and improve the prognosis of hemorrhagic stroke in mice (Karuppagounder et al., 2018).

### Others

Naotaifang reduces TFR1 expression, ROS and iron accumulation, and neurobehavioral scores in a rat model of acute cerebral artery occlusion (Lan et al., 2020). Moreover, Carvacrol reduces the tissue lipid peroxide levels to protect gerbils from ischemia-reperfusion injury (Guan et al., 2019).

## Ferroptosis in Neurotrauma

### The Mechanism of Ferroptosis in Neurotrauma

Post-TBI cell death is a cause of neurological deficits and death (Stoica and Faden, 2010; Anthonymuthu et al., 2018). After brain trauma, there is overexpression of lipid peroxidase, including cyclooxygenase and LOX. One of the earliest mechanical injury events is excessive free fatty acid release. There was a significant increase in free fatty acids in the brain within 5 min after TBI, which remained high at 24 h after the injury (Dhillon et al., 1994). There is a post-TBI increase in the biomarkers of iron-induced death. Further, LOX inhibition in ferroptosis can protect HT22 neurons from *in vitro* TBI and promote tissue neuroprotection and PE oxidation after control cortex injury (Kenny et al., 2019). Therefore, ferroptosis prevention in brain trauma strengthens the defense system, removes oxidized membrane lipids, prevents 15LOX/PEBP1 complex formation, and reduces the membrane lipid dioxide oxidation rate (Anthonymuthu et al., 2018).

### Ferroptosis Inhibitors in Neurotrauma

First, the PEBP1/15LOX complex in cortical and hippocampal neurons is a potential treatment target for brain trauma. This is based on the balance between 15-LOX-produced PE-PUFA-OOH metabolites and GPX4 reduction to hydroxyl metabolites in ferroptosis (Wenzel et al., 2017). Therefore, specific inhibitors targeting the PEBP1/15-LOX complex could serve as a novel treatment strategy for ferroptosis (Jameson et al., 2014). Similarly, Baicalein inhibits ferroptosis and improves post-TBI behavioral outcomes (Andersen et al., 2019; Kenny et al., 2019). Additionally, N-acetylcysteine has a protective effect on GSH in adult, but not pediatric, patients with TBI (Amen et al., 2011; Hoffer et al., 2013).

## Differences and Relationships Among Various Cell Death Mechanisms in Neurological Diseases (Table 1)

Different cell death mechanisms, apoptosis, necrosis, autophagy and ferroptosis occur in neurological diseases (Guiney et al., 2017). Each mechanism has typical morphology, regulators, and inhibitors (Table 1). Assessment of intracellular proteases in neurological diseases by Yagami et al. (2019) indicated that neuronal cell death is induced by the generation of ROS, including H_2_O_2_; increased intracellular calcium levels; activation proteases (caspase, calpain, cathepsin), and inactivation/activation of kinases.

**TABLE 1:** Differences and relationships among various cell death mechanisms in neurological diseases.

**Cell death**	**Differences**						**Relationships**
		**Morphology**	**Regulators**	**Inhibitors**	**Related neurological disease**	**Immunity**	
Programmed cell death	Apoptosis	cell shrinkage, plasma membrane blebbing, karyorrhexis, chromatin condensation, and DNA fragmentation	Caspase Bax, Bak AIF 1 P53	Bcl-2	PD (Guiney et al., 2017) Stroke (apoptosis in the penumbra) (Yagami et al., 2019) AD (Sobottka et al., 2008) ASL (Friedlander et al., 1997) HD (Gil and Rego, 2008)	Cleared by macrophages or other phagocytic cells (Proneth and Conrad, 2019)	Biochemical substances related to ferroptosis metabolism inhibit the execution of apoptosis (Dixon, 2017).
	Ferroptosis	Atrophy of mitochondria rounded-up cell body and changed mitochondrial morphology and cristae structure	Fe Lipid ROS	Iron chelators, GPX4, Fer-1, Lip-1, FSP-1	PD (Guiney et al., 2017) AD (Cong et al., 2019) HD (Skouta et al., 2014) Stroke (Zille et al., 2017) Neurotrauma (Hoffer et al., 2013)	Immunogenicity	Ferroptosis in neurological diseases induced by different targets.
	Autophagy	Autophagosome, double membrane vesicle formation	Calpain Atg LAMP2	3-MA	PD (Guiney et al., 2017) ICH (Zille et al., 2017) Stroke (Adhami et al., 2006) ALS (Bruijn et al., 1997) HD (Gil and Rego, 2008)	N/A	NCOA4-mediated ferritinophagy is an autophagic phenomenon which induce ferroptosis by degradation of ferritin.
Accidental cell death	Necrosis	Swelling of cytoplasm and organelles, karyolysis and disruption of plasma membrane	Cathepsin RIP1 RIP3	Nec-1	PD (Guiney et al., 2017) Stroke (necrosis in the core of the injury) (Christofferson and Yuan, 2010) ICH (Li et al., 2018)	Immunogenicity	Ferroptosis may be a regulator of necrotic cell death (Martin-Sanchez et al., 2018).

**AIF1, apoptosis-inducing factor 1; Atg, autophagy-related genes; 3-MA, 3-methyladenine; Nec-1, necrostatin-1; RIP, receptor interacting protein.**

First, the morphological characteristics of apoptotic neuron cells include cell shrinkage, plasma membrane blebbing, karyorrhexis, chromatin condensation, and DNA fragmentation. Apoptosis, which is a common programmed cell death mechanism, has been demonstrated in the penumbra of patients with stroke (Yagami et al., 2019) and neurodegenerative diseases, including PD, AD (Sobottka et al., 2008), ALS (Friedlander et al., 1997), and Huntington disease (HD) (Gil and Rego, 2008). Further, patients with PD present with elevated levels of proapoptotic protein Bax and activated caspase in dopaminergic neurons (Guiney et al., 2017). *In vitro* cell experiments have shown that caspase-activated upstream apoptotic mechanisms are not involved in cellular ferroptosis; moreover, GSH-depleted cells cannot properly activate caspase (Dixon et al., 2012). Accordingly, Dixon (2017) suggested that ferroptosis-related biochemical substances inhibit apoptosis.

Second, necrosis is characterized by the swelling of cytoplasm and organelles, karyolysis and plasma membrane disruption in collagenase-induced mouse ICH models observable using transmission electron microscope (Li et al., 2018). Necrosis, which refers to accidental cell death (Yagami et al., 2019), is observed in early stage stroke injury (Christofferson and Yuan, 2010). In addition, necroststin-1, the necrosis inhibitor, reduces the toxicity of 6-hydroxydopamine (6-OHDA) in the PD model (Guiney et al., 2017). Ferroptosis may be a regulator of necrotic cell death (Proneth and Conrad, 2019); specifically, it may induce secondary upregulation of necrotic mechanical components and exacerbate tissue damage (Martin-Sanchez et al., 2018).

Third, autophagy has been observed in neurological diseases. Three days after ICH, autophagosome and double-membrane vesicle formation have been observed in the mouse ICH model (Li et al., 2018). Moreover, the morphological characteristics of autophagy have been observed in patients with PD (Guiney et al., 2017). In adult mice with carotid artery occlusion and hypoxia, stroke causes reperfusion deficits and autophagic/lysosomal cell death in the brain (Adhami et al., 2006). In addition, autophagosomes appear in the neurons of patients with ALS (Bruijn et al., 1997) and HD (Gil and Rego, 2008). Notably, it induces ferroptosis, which leads to ferritinophagy (Vanden Berghe et al., 2014). NCOA4-mediated ferritinophagy is an autophagic phenomenon that induces ferroptosis via ferritin degradation (Hou et al., 2016). Under ferroptotic conditions, ferritin is degraded by autophagy, which preserves the labile iron pool and makes the cell more sensitive to ferroptosis (Gao et al., 2016). Further, autophagy may share key regulators with ferroptosis (Santana-Codina and Mancias, 2018).

Taken together, the relationship among apoptosis, necrosis, autophagy and ferroptosis is delicate and complex in neuronal and surrounding glial cells, with each mechanism having its corresponding time windows. In addition, cell death forms vary across different brain regions with different neuronal sensitivity. For example, the ventral region is more sensitive to PD neurodegeneration in the substantia nigra area (Guiney et al., 2017). In addition, given that pathology caused by tissue damage and degeneration is related to inflammation, the immune response and involved cells could be used to distinguish among the different cell death mechanisms (Green et al., 2009). Apoptotic cells are cleared by macrophages or other phagocytic cells based on the plasma membrane integrity (Proneth and Conrad, 2019). Contrastingly, in necroptosis and ferroptosis, the release of damage-associated molecular patterns through the ruptured plasma membrane triggers the innate immune system in brain tissues with high immunogenicity (Dixon, 2017; Proneth and Conrad, 2019). Regarding therapy, Fer-1 combined with different cell death inhibitors allows better prevention of hemoglobin-induced cell death in organotypic hippocampal slice cultures and human pluripotent stem cell-derived neurons than any of the inhibitors alone. In a collagenase-induced mouse ICH model, a combination of Fer-1, caspase3 inhibitor, and necrostatin-1 can reduce cell death more than any of the inhibitors alone (Li et al., 2017).

## Summary

The related ferroptosis mechanisms in neurological diseases are summarized in Figure 1 and the recent drugs that target ferroptosis for neurological disease treatment are summarized in Table 2. There are several prospects regarding ferroptosis in the nervous system. First, there is a need for chemical probes or biomarkers capable of elucidating the ferroptosis mechanism to determine its role in the nervous system given the quick cell death occurrence and difficulty in obtaining appropriate neurons (Guiney et al., 2017). In contrast with other cell death mechanisms, mitochondrial atrophy (Zille et al., 2017), as well as immunity-related cells and factors (Proneth and Conrad, 2019), may be potential markers. Moreover, cyclooxygenase (Li et al., 2017) and lipid peroxides can be assessed as potential chemical probes or biomarkers (Dixon, 2017; Stockwell et al., 2017). It is showed in the latest report that nanoparticles with adjustable size, charge, and targeting ligands show high specificity for the brain, which may provide a reference for the design of new anti- ferroptotic nanomedicines in the future (Xu et al., 2020). Second, since ferroptosis is a cell sabotage mechanism, it should be assessed as a potential adaptive change in the body’s response to stimuli (Dixon, 2017). Third, redox phosphatidyl liposomes in the ferroptosis process involve, PE- and PUFA-PEox as substrates and redox reactions products and catalytic or regulatory proteins, such as 15-LOX, PEBP1, and GPX4. The interactions among these liposome components could elucidate relevant pathogeneses and they could be assessed as potential targets for drug therapy (Wenzel et al., 2017). Fourth, the use of LOXBlock-1 with tissue plasminogen activator significantly reduces bleeding, which suggests that this combination could be a useful treatment strategy for ischemic stroke. Further research is required to determine whether simultaneous administration is required, as well as the appropriate time window (Yigitkanli et al., 2013). Fifth, while assessing the role of the various ferroptosis inhibitors, attention should be paid to the combined use of other cell deaths inhibitors and different drug delivery methods.

**TABLE 2:** Potential drugs that target ferroptosis for neurological disease treatment.

**Ferroptosis inhibitors**	**Neurotrauma**	**Stroke**		**Neurodegeneration**			
		**ischemic**	**hemorrhagic**	**PD**	**AD**	**HD**	**ALS**
Iron chelators	N/A	DFO (Weiland et al., 2019)		DFP (Chen and Xie, 2020) Clioquinol (Shi et al., 2020)	PBT2 (Ward et al., 2014)		N/A
Lipid ROS Inhibitors	XJB-5-131 (Ji et al., 2012) Luteolin (Xu et al., 2014) NAC (Hoffer et al., 2013)	Lip-1 (Tuo et al., 2017) Fer-1 (Li et al., 2017) NAC (Khan et al., 2004) Baicalein (van Leyen et al., 2006) ML351 (Inhibitor of 12/15-LOX) (Rai et al., 2014) Trolox (Zille et al., 2017) LOXBlock-1 (Yigitkanli et al., 2013) Tat Selpep (Alim et al., 2019)	GPX4 (Zhang Z. et al., 2018) Tat Selpep (Alim et al., 2019) N-acetylcysteine (NAC) (Karuppagounder et al., 2018) Fer-1 (Li et al., 2017) Ebselen (Azad and Tomar, 2014)	GPX4 (Chen et al., 2015) FSP1 (Chen and Xie, 2020) Cu II (Southon et al., 2020)	Hydroxylated Chalcones (Cong et al., 2019) GPX4 (Yoo et al., 2010) PD146176 (Inhibitor of 12/15-LOX) (Di Meco et al., 2017) α-Lipoic Acid (Zhang Y. H. et al., 2018)	NAC (Wright et al., 2015) Fer-1 (Skouta et al., 2014)	Cu II (Southon et al., 2020)
				Lip-1 (Hambright et al., 2017) Fer-1 (Skouta et al., 2014) Mithramycin (Ratan, 2020)			
				XJB-5-131 (Krainz et al., 2016) CoQ10 (Weiland et al., 2019) Tecfidera (Sejbaek et al., 2019)			
				Radical trapping antioxidant (Li et al., 2019) Gastrodin (Jiang et al., 2020)			
HIF-PHD inhibitors	N/A	peptide HIF prolyl 4-hydroxylase inhibitors (Siddiq et al., 2005) Dimethyloxalylglycine (DMOG) (Nagel et al., 2011) DHB (Karuppagounder and Ratan, 2012)	Adaptaquin (Karuppagounder et al., 2016) Hypoxia-inducible factor prolyl hydroxylase inhibition (Karuppagounder and Ratan, 2012)	N/A	Adaptaquin (Karuppagounder et al., 2016)	N/A	N/A
Others	Baicalein (Andersen et al., 2019)	Naotaifang (Lan et al., 2020) Carvarol (Guan et al., 2019)	N/A	HPL (Gouel et al., 2017) PKC inhibitors-Bis III (Do Van et al., 2016)	N/A	N/A	HPL (Gouel et al., 2017)
				1-HPPL (Nebie et al., 2019)			
	Vit E/α-tocopherol (αToc) (Matsushita et al., 2015)						

## Author Contributions

J-XR and XS searched the literature and drafted the manuscript. X-LY, Z-NG, and YY critically revised the manuscript. All authors contributed to the article and approved the submitted version.

## Conflict of Interest

The authors declare that the research was conducted in the absence of any commercial or financial relationships that could be construed as a potential conflict of interest.

## Abbreviations

AD: Alzheimer’s disease
CNS: Central nervous system
DFO: Deferoxamine
DFP: Deferiprone
Fer-1: ferrostatin-1
Gln: glutamine
GPX4: Glutathione peroxidase-4
GSH: glutathione
HD: Huntington’s disease
HIF: hypoxia-inducible factor
ICH: intracerebral hemorrhage
Lip-1: liproxstatin-1
12/15-LOX: 12/15-lipoxygenases
NCOA4: Nuclear receptor coactivator 4
PD: Parkinson’s disease
PE: phosphatidylethanolamines
PHDs: prolyl-hydroxylases
PUFA: polyunsaturated fatty acid
ROS: reactive oxygen species
TBI: traumatic brain injury
TF: transferrin
TFR1: transferrin receptor 1.
